# The First Reported Pediatric Case of Primary Myoepithelial Carcinoma Involving the Whole Lung: Surgical Radical Treatment and Prosthesis Implant

**DOI:** 10.1055/s-0040-1713766

**Published:** 2020-08-19

**Authors:** Claudia Filisetti, Tiziana Russo, Andrea Pansini, Claudio Vella, Camilla Viglio, Giovanna Riccipetitoni

**Affiliations:** 1 Department of Pediatric Surgery, “V.Buzzi” Children Hospital, University of Pavia, Milano, Italy

**Keywords:** pediatric surgery, myoepithelial carcinoma, pediatric, cancer

## Abstract

Primary myoepithelial carcinoma of the lung (PMC-L) arising from the bronchial glands in lower respiratory tract is exceedingly rare. Thus far, few cases in adults and only one in a pediatric patient have been recorded. To our knowledge, this is the first report of PMC-L successfully removed in a child, focusing on the importance of multidisciplinary primary surgery for the treatment of this tumor. A 7-year-old girl was admitted for persistent cough and fever; she was unresponsive to oral antibiotics. Chest radiography showed loss of volume of left lung sustained by almost total atelectasis. After routine clinical investigations, she was referred for computed tomography scan and magnetic resonance imaging that documented the presence of a mass occupying the entire left upper lobe, infiltrating the pulmonary hilum (main bronchus, pulmonary artery, superior pulmonary vein, and pericardium). After multidisciplinary evaluation, the histopathologic diagnosis of PMC-L was established using ultrasonography-guided transthoracic core needle biopsy and bronchoscopic biopsies. She was then subjected to left pneumonectomy under extracorporeal circulation and positioning of a thoracic expander filled with 200 mL of saline solution. The postoperative course was uneventful. With TREP (very Rare Tumor in Pediatric Age) consent radiotherapy was performed (61.2 Gy). At the 10-month follow-up, the patient was alive, breathing normally without any oxygen support, without recurrence of PMC-L or metastasis, and without any chest deformity. To our knowledge, this is the first case where a pediatric patient was successfully operated for PMC-L involving the whole lung. Extracorporeal circulation enabled us to perform radical primary surgery. Prosthesis implant not only maintained normal chest expansion but also allowed focused radiotherapy, thus enabling us to prevent damage to vital organs.

## Introduction

Epithelial–myoepithelial tumors are rare neoplasms that originate from the salivary glands and comprise 1% of all primary tumors. Epithelial–myoepithelial carcinoma was first described in the salivary glands in 1972. The World Health Organization has classified this carcinoma as a malignant tumor composed of variable proportions of two cell types that typically form duct-like structures. 1 2 These tumors are extremely rare in the lung. They include mucoepidermoid carcinoma, adenoid cystic carcinoma, acinic cell carcinoma, oncocytoma, epithelial–myoepithelial carcinoma, benign myoepithelioma, and mixed tumors. 3 4 5 Primary pulmonary myoepithelial carcinoma is a rare neoplasm that is thought to arise from the submucosal bronchus. These glands are considered to be a form of minor salivary glands. Primary pulmonary myoepithelial carcinoma is a relatively lower grade malignant; however, it shows high rates of distant metastasis. Owing to the low incidence, there is no guideline regarding the optimal therapeutic strategy. 6 Surgical treatment is the only available therapeutic option. Thus far, only two pediatric cases have been recorded. 7 8 To our knowledge, the present case is the first pediatric case treated with surgery and radiotherapy.

## Case Report

**Fig. 1:**
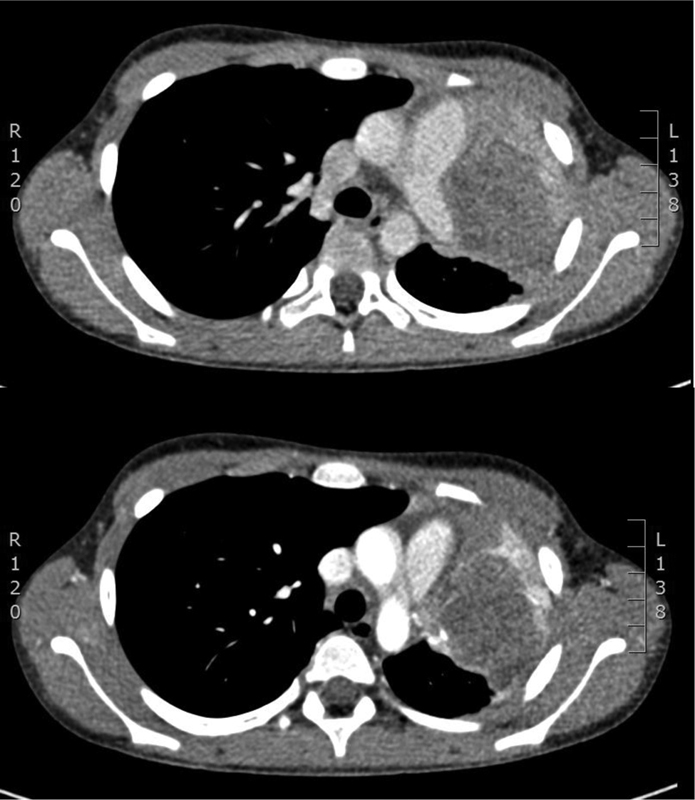
Preoperative computer tomography showing the tumor.

Chest radiography was performed for a 7-year-old girl with persistent cough and fever who was unresponsive to oral antibiotics and showed loss of volume of left lung sustained by almost total atelectasis. The patient was then admitted to the pediatric ward to begin intravenous antibiotic therapy. Serological tests for *Chlamydia pneumoniae* , *Mycoplasma pneumoniae* , and QuantiFERON test for tuberculosis were negative. A computed tomography (CT) scan showed an extended mass that occupied the entire upper lobe with axial dimensions of 70 × 47 mm and assumed discreet enhancement in all the study phases. The bronchi for the upper lobe were no longer patent with endobronchial projection affecting the middle third of the main left bronchus. The mass caused posterior dislocation of the pulmonary arterial vascular axis and inferior dislocation of the superior pulmonary vein. Based on the radiological reports, the patient was referred to our pediatric surgery unit. To better define the characteristics of the lesion, a magnetic resonance imaging was performed. The pictures confirmed the presence of an expansive pulmonary lesion with a probable origin from the upper left lobe with transverse diameters of 57 × 45 mm and longitudinal extension of 85 mm. The lesion infiltrated the pulmonary hilum and invaded the bronchial structures up to the left main bronchus ∼2 cm from the carina. It appeared adherent to the common trunk of the pulmonary artery and to the pericardium at the level of the left atrium and ventricle with infiltration of the superior pulmonary vein. The mass appeared to be surrounded by a thin rim of consolidated lung parenchyma; therefore, it does not seem to infiltrate the thoracic wall ( Fig. 1 ). For a complete diagnostic work-up, positron emission tomography–CT was performed; the findings showed softened uptake of the tracer at the voluminous expansive formation that tended to accentuate along the margins. Echocardiography revealed a 7-mm ostium secundum atrial defect with left to right shunt and deformity of the left ventricular cavity, with preserved systolic function. The deformity was attributable to the mass. All the tumor markers were negative. After multidisciplinary evaluation, ultrasonography-guided transthoracic core needle biopsy (22 Gauge) and bronchoscopic biopsies were performed to establish the diagnosis. Histopathologic examinations revealed the presence of medium–small-sized cells neoplasia, ovoid and plasmacytoid, with eosinophilic cytoplasm, lacking of evident cytological atypia with solid growth or in large nests alternating with myxoid and dense collagenous stroma. Immunohistochemical reactivity was positive for cytokeratine (CK) AE1AE3, p63, epithelial membrane antigen (EMA), S100, β-catenin, SOX10, and terminal deoxynucleotidyl transferase (TDT). The morphological and immunophenotypic data were more suggestive of myoepithelioma. However, the immunocytochemical expression of TdT did not exclude the possibility of thymic neoplasm. Fluorescence in situ hybridization analysis showed the absence of translocation of *FUS* gene in 16p11, *EWS* gene in 22q12, and *SS18* gene in 18q11.2. The case was discussed among pediatric surgeons, oncologists, and radiologists who concluded that the nature of the cancer was not sensitive to neoadjuvant treatment, thus indicating elective radical surgery.

### Surgery

**Fig. 2:**
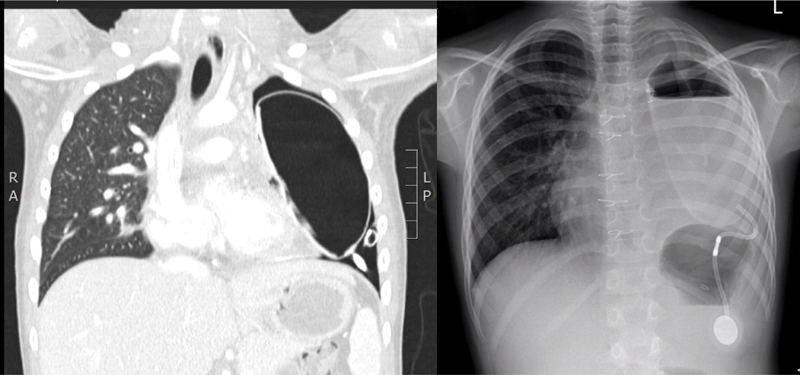
Postoperative imaging.

After the performance of sternotomy and opening of the pleura bilaterally, the left thoracic mass was revealed. The mass infiltrated the entire left lung and was attached to and infiltrated the pericardium, the left pulmonary artery and veins, as well as the left bronchus. We proceeded to release the left lower lobe by making selective distal ligation of the pulmonary arterial and venous vessels and of the left lobar bronchus. The lobectomy was completed with a mechanical stapler. The lower lobe appeared infiltrated by the mass at the level of the fissure. After resecting the lingular lobe, it was necessary to proceed under extracorporeal circulation to isolate the left pulmonary artery and veins. We isolated the pulmonary artery trunk as well as tied and dissected the Botallo's duct and the left branch of the pulmonary artery. The two left pulmonary veins and the main left bronchus were dissected and sutured. The mass was isolated from the remaining pericardium, from the vagus nerve, and from the phrenic nerve. Once the left pneumonectomy was completed and hemostasis was achieved, the interatrial defect was surgically closed, and a temporary pacemaker was implanted. A thoracic expander of 11 × 6 cm was applied and filled with 100 mL of saline solution. The expander reservoir connector was placed outside of the chest wall, and the reservoir was placed in a subcutaneous pocket. A left thoracic drain was placed dorsal to the expander. The postoperative course was uneventful. The temporary pacemaker was removed after 5 days, and the drain was removed after 8 days. The expander was gradually filled up to 200 mL with saline in three different infusions over 3 weeks ( Fig. 2 ). The patient was discharged on postoperative day 18. Histopathological analysis confirmed the presence of primary myoepithelial carcinoma of the lung (PMC-L) that infiltrated the bronchial wall up to the mucosa, the peribronchial fibroadipose tissue with vascular perineural–neural invasion, the pulmonary parenchyma, the visceral pleura, the pericardium, and a peribronchial lymph node with embolic metastasis. In view of the rarity of this neoplasm, the TREP group (“Rare Tumors in Pediatric Age”) means rare tumors in pediatric age group was consulted to set up a plan for the follow-up care. The patient was then subjected to radiotherapy with rapid arc technique on the left thoracic wall in 34 diaphragmatic thickening fractions, 61.2 Gy in total. At the 14-month follow-up, the patient was alive and was breathing normally without oxygen support. Neither recurrence of PMC-L nor metastasis occurred, and no chest deformities were observed.

## Discussion

Myoepithelial carcinoma primarily arises from the salivary glands, rarely from the soft tissue. 9 10 11 12 PMC-L was described for the first time in 1998; therefore, we identified 12 cases in adult patients and 2 in pediatric patients. Treatment of the primary pulmonary myoepithelial carcinoma involves complete surgical resection followed by radiotherapy. In case of metastatic disease, chemotherapy and radiotherapy are the standard treatments, even if there is insufficient information to define the survival and efficacy of these treatments. Some reports have shown that this tumor is not sensitive to radiotherapy and chemotherapy. Recommendations regarding chemotherapy or radiation, either pre- or postoperatively, are difficult to formulate. Close long-term follow-up of this patient may help us elucidate the natural history of this rare tumor. There are only two pediatric case described in the literature. The first patient was affected by an advanced metastatic disease with the disappearance of pulmonary opacity spontaneously without any treatment 7 ; the second case was a relatively small PMC-L in a 7-year-old boy treated surgically. 8 In our case, the biopsy made it possible to establish the diagnosis. In light of the clinical picture, disease stage, and available reports, we decided to treat the disease surgically. One challenge in the case of total pneumonectomy in pediatric patients is the postpneumonectomy syndrome. In our patient, we opted for the placement of a saline-filled tissue expander into the pleural space, as reported in previous experiences to avoid postpneumonectomy syndrome. Postpneumonectomy syndrome occurs in ∼2% of the patients undergoing pneumonectomy for a variety of lung pathologies. It involves excessive mediastinal shift and rotation toward the empty hemithorax from where the patient's diseased lung was previously removed. This results in airway and esophageal obstruction that may lead to death. 13 In our case, the use of the expander was a better treatment from the radiotherapeutic point of view, as reported in other conditions wherein the insertion of a spacer has led to advancements in the field of radiotherapy. 14 15

## Conclusion

PMC-L is exceedingly rare. To our knowledge, our case represents the first pediatric case with surgical treatment of PMC-L that involved the whole lung. Surgery is the main treatment for operable patients. In our case, surgical excision was successful and can be expected to be curative.

## Notes

New Insights and the Importance for the Pediatric Surgeon Report the surgical approach for an extremely rare neoplasm (primary myoepithelial carcinoma of the lung) for which there are no precise guidelines in the literature.
